# Prevalence of depression, anxiety in China during the COVID-19 pandemic: an updated systematic review and meta-analysis

**DOI:** 10.3389/fpubh.2023.1267764

**Published:** 2024-01-05

**Authors:** Xiang Bin, Ke-Yi Qu, Yu-Hao Wang, Li Chen, Yan-Jie Xiong, Jin Fu Wen, Hua-Bo Wei, Tan Bing, Chun-Yan Dan, Jia-Quan Zhu

**Affiliations:** ^1^Department of Otolaryngology, Fengdu County People's Hospital, Chongqing, China; ^2^Department of Stomatology, Fengdu County People's Hospital, Chongqing, China; ^3^Shandong Academy of Medical Sciences, Shandong First Medical University, Shandong, China; ^4^Department of Pharmacy, Fengdu County People's Hospital, Chongqing, China; ^5^Institute of Cardiovascular Endocrinology of Shandong First Medical University, Shandong Academy of Medical Sciences, Shandong, China; ^6^Department of Human Resources, Fengdu County People's Hospital, Chongqing, China

**Keywords:** COVID-19, depression, anxiety, China, systematic review, meta-analysis

## Abstract

**Background:**

Mental health risks associated with the aftermath of the COVID-19 pandemic are often overlooked by the public. The aim of this study was to investigate the effects of the COVID-19 pandemic on depression and anxiety disorders in China.

**Methods:**

Studies were analyzed and extracted in accordance with the PRISMA 2020 flowchart. The studies were screened and extracted using electronic databases including PubMed, Web of Science, Embase, Cochrane Library, and ClinicalTrials.gov according to the predefined eligibility criteria. The Cochrane Review Manager software 5.3.1 was used for data analysis and the risk of bias assessment.

**Results:**

As of 2023, a total of 9,212,751 Chinese have been diagnosed with COVID-19 infection. A total of 913,036 participants in 44 studies were selected following the eligibility criteria, the statistical information of which was collected for meta-analysis. The pooled prevalence of depression and anxiety were 0.31 (95% CI: 0.28, 0.35; *I*^2^ = 100.0%, *p* < 0.001) and 0.29 (95% CI: 0.23, 0.36; *I*^2^ = 100.0%, *p* < 0.001), respectively. After performing a subgroup analysis, the prevalence of depression among women, healthcare workers, students, and adolescents was 0.31 (95% CI: 0.22, 0.41), 0.33 (95% CI: 0.26, 0.44), 0.32 (95% CI: 0.26, 0.39), and 0.37 (95% CI: 0.31, 0.44), respectively.

**Conclusion:**

The prevalence of depression and anxiety among the Chinese was overall high. Monitoring and surveillance of the mental health status of the population during crises such as sudden global pandemics are imperative.

**Systematic review registration:**

https://clinicaltrials.gov/, identifier [CRD42023402190].

## 1 Introduction

The novel coronavirus disease (COVID-19), which is believed to have originally erupted in Wuhan, China, in December 2019, was declared an international public health emergency by the World Health Organization (WHO) in January 2020, followed by an emergency declaration of the outbreak as a global pandemic by WHO on 11 March 2020 (1). Although COVID-19 was initially recognized as a respiratory disease, the variant strain of SARS-CoV-2 has the capability to destroy many organ systems (2, 3). Early research revealed an increasing risk of long COVID-19 sequelae after the second or third infections, even in double-vaccinated and triple-vaccinated individuals (4, 5). Suicide is the most severe sequelae of untreated COVID-19 psychiatric disorders (6, 7).

To combat the rapid spread of COVID-19 among the general population, the Chinese government initially imposed a complete lockdown in Wuhan and then gradually imposed lockdowns in other cities (8). Initially, the Chinese public was understanding and appreciative of the Chinese government’s efforts to prevent the spread of infection, whereas with the increasing isolation time and infection number, emotional symptoms such as panic, depression, and anxiety continued to rapidly breed and spread among ordinary people. These psychological effects are thought to be related to fear of infection, helplessness after being quarantined, unemployment due to the blockade, market supply interruption due to the transportation blockade, negative media coverage, and social discrimination due to COVID-19 infection (9–11). Increasing evidence has suggested that the psychological impact of the COVID-19 pandemic may have a long-term effect on one’s overall mental health (12–14).

Since the outbreak, public health authorities and healthcare researchers have focused heavily on the biological and physiological impacts of COVID-19, with little attention paid to its impact on mental health. During the COVID-19 pandemic, the psychological wellbeing of students, women, and healthcare workers was often seriously threatened due to their occupational characteristics but often overlooked (15–17). Population prevalence meta-analyses in China, the origin of the epidemic, home to approximately one-fifth of the world’s population, are an important reference for global evidence-based medicine, yet data on the mental health impact of the COVID-19 pandemic on the general population remain inadequate.

We conducted a systematic review and meta-analysis to synthesize the accumulated research on mental disorders and COVID-19. The objective was to deeply quantify the mental health impact (depression and anxiety) and prevalence of COVID-19 in China during the whole period of the COVID-19 pandemic. It is believed that the pooling of data will bring more attention to the mental health issues resulting from global public health event pandemics such as COVID-19 and provide theoretical support for the introduction of preventive policies for mental health issues that may result from new global public health events in future. There is no doubt that although the WHO no longer classifies COVID-19 as a public health emergency, our study, similar to the global pandemic SARS-CoV-2 at the beginning of this century, may still provide an important complementary theoretical basis for the adoption of preventive measures for future global public health emergencies resulting in affective disorders for quite some time. After all, no one can be certain that the COVID-19 incident is the endpoint of a global public health event, and the SARS coronavirus is an example.

## 2 Methods

This systematic review was conducted following the PRISMA 2020 guidelines (18). The protocol for the review was registered and published in PROSPERO (ID: CRD 42023402190).

### 2.1 Search strategy

Articles were retrieved through a systematic search of PubMed, Web of Science, Embase, Cochrane Library, and ClinicalTrials.gov. To broaden the search scope and improve accuracy, a selected search strategy combining keywords and subject terms was used to optimize the results. Briefly, the initial search strategy mainly included keywords such as COVID-19, depression, anxiety, China, or Chinese, and the detailed search strategies are presented in Supplemental File S1. The initial filtering method was as follows: Initially, titles were filtered for potentially relevant articles, followed by an elaborate screening of abstracts to confine the search. Full texts of the article were filtered and shortlisted based on eligibility and relevance to the topic. Two independent reviewers reviewed all the articles to be included, and Cohen’s kappa coefficient valuation was used to assess the consistency of the two reviewers at each stage of the process for the inclusion of the articles, and when the reviewers agreed with the inclusion of a particular piece of literature, it was marked accordingly (yes “1” and no “0”).

### 2.2 Eligibility criteria

The inclusion criteria were as follows: (a) studies in which the prevalence of depression, anxiety, or a combination of the two was evaluated as the main outcome; (b) the research field was the completed or ongoing research on the mental health of COVID-19 during the pandemic in China; (c) inclusion was restricted to studies conducted in China, and the entire study population was limited to Chinese only; (d) only cross-sectional surveys were included; and (e) the ultimate result of the data was presented as a percentage or frequency. However, studies that meet any of the following criteria were excluded: (a) non-cross-sectional surveys; (b) data in any language other than English; (c) depression or anxiety was not assessed as a major outcome; (d) data were not quantified by statistical scales; (e) research results expressed in addition to frequency and percentage, such as mean and standard deviation; and (f) unavailable full-text articles.

### 2.3 Data extraction

Two reviewers independently screened the titles and abstracts according to the same selection criteria and extracted the data of potentially relevant studies based on a predefined qualification criteria form (MS-Excel). Resolution of disagreements between the two review authors was done based on a review of eligibility and discussion with a third independent reviewer until a consensus was reached. After eliminating duplicate articles and data, we formulated a data extraction strategy flow chart following the PRISMA guidelines. Briefly, the following information was extracted from studies included in the systematic review: author, year of publication, research type, number of participants, response rate, gender ratio, age (denoted as “MD ± SD”), region, scale type, participant type, and outcome indicators (denoted as “%”). “NR” is denoted as the presence of unclear data in the extracted articles.

### 2.4 Risk of bias assessments

Briefly, two strategies were chosen to assess the risk of bias in the included studies. First, we carefully assessed the risk of bias in each included study through the Cochrane risk assessment tool, which is a mature and widely used tool for article quality assessment in meta-analysis. Specifically, this tool is widely used due to its powerful analysis function, which includes assessing the representativeness, methodology, data reliability, and authenticity of the study in detail. Furthermore, we used the Newcastle-Ottawa Quality Assessment Scale (a tool exclusively used for risk assessment of cross-sectional studies) to minimize the risk of bias (19). The score for high-quality studies was specified as 3 and above; in other words, studies with a score ≥ 3 were considered low-risk studies. The scoring of all risk assessment processes was completed by three independent authors simultaneously, and in case of disagreement, the fourth author was involved in consultations to reach a consensus.

### 2.5 Statistical analysis

Review Manager 5.3 (RevMan 5.3) was utilized to conduct the meta-analysis. Since the prevalence rate was readily provided in all studies, it was used for data analysis instead of the log odds ratio. Cochran’s *Q*-test (chi-square) and *I*^2^ statistics were chosen to determine heterogeneity in prevalence estimates. The heterogeneity was considered low, moderate, and high if the cutoff points for *I*^2^-values of 25, 50, and 75% or more were found (20). The random effects model was chosen as it accounts for variance in effect sizes between studies. Pooled prevalence with their 95% confidence intervals (95% CI) was considered as the measure of effect. The effect size and 95% CI for each study were presented as forest plots. Moreover, Arc Map 10.8 was utilized to model the COVID-19 pandemic in various regions of China.

Subgroup analysis of the primary outcome (prevalence of depression) was performed using RevMan 5.3, which summarizes 34 studies investigating the effect of genders and population types on the prevalence of depression. Sensitivity analysis was used to assess the alteration in pooled effect sizes. Specifically, it was performed by removing individual studies one by one and conducting a meta-analysis after removing each study. This cumulative analysis was widely used to estimate the effect of the largest study on the pooled effect size.

## 3 Results

### 3.1 Studies selection

**Figure 1:**
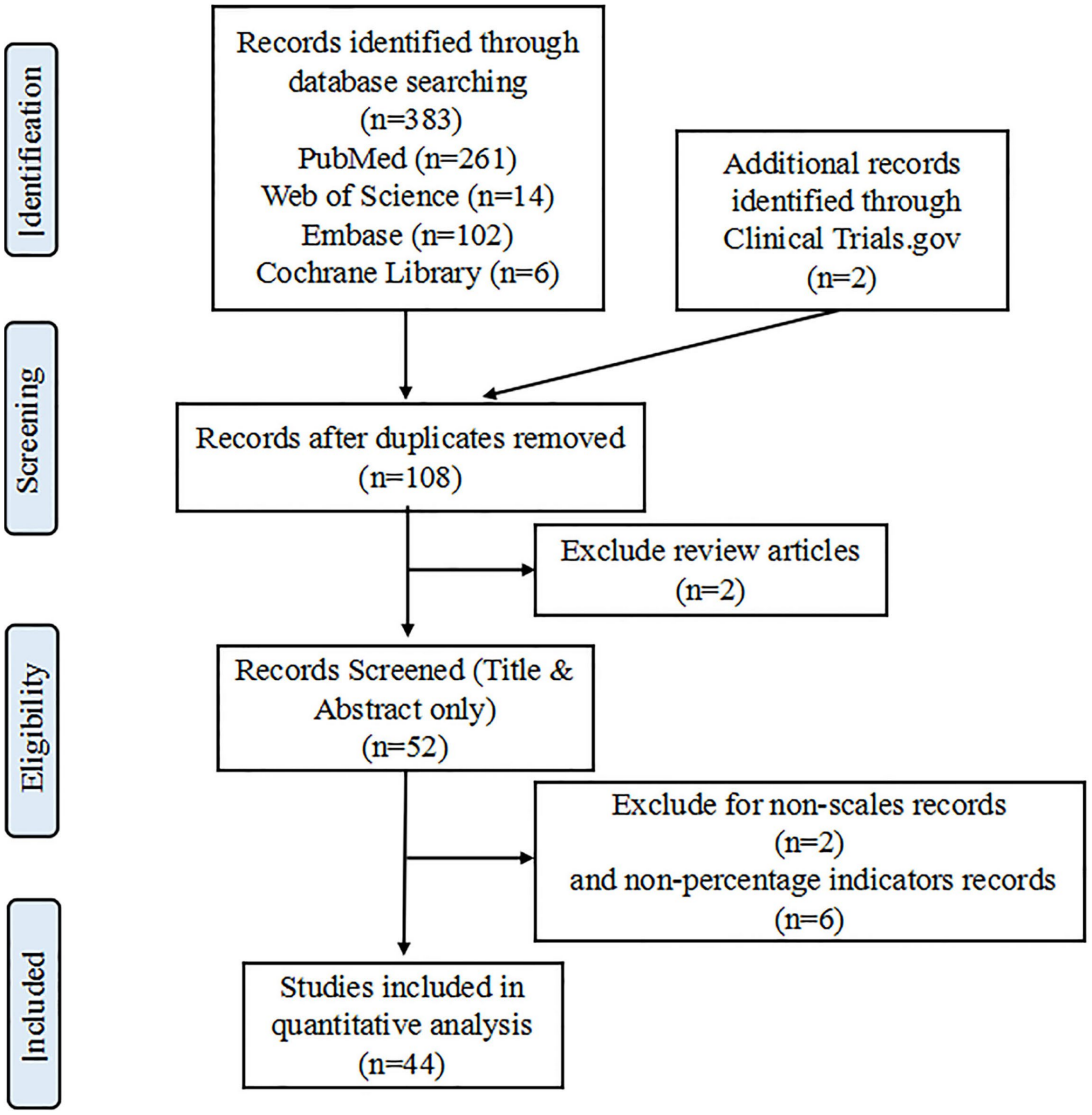
PRISMA flow diagram of the systematic review and meta-analysis selection process.

The initial search yielded 385 articles published between 1 December 2019 and 1 March 2023, from which 277 duplicates were removed, following which 108 full-text articles were assessed for eligibility. After screening titles and abstracts, 106 articles were eligible for full-text assessment, of which 52 were included. A total of 44 articles were then finalized and included in the quantitative meta-analysis. Interestingly, during the COVID-19 pandemic in China, no articles were found that included anxiety prevalence as an evaluated outcome alone; all relevant articles jointly assessed the prevalence of depression with anxiety and even stress prevalence among the population. Moreover, two independent reviewers reached a fair degree of consistency (Cohen’s kappa coefficient = 0.23) at the initial stage of the inclusion of the literature (*N* = 108), a moderate level of consistency (Cohen’s kappa coefficient = 0.66) after carefully reading the abstract (*N* = 52), and a strong consistency (Cohen’s kappa coefficient = 0.86) after carefully checking the inclusion criteria (*N* = 44). Disagreements that existed at all stages were eventually completely eliminated in consultation with a third senior reviewer, and further selection was only possible once disagreements at each stage had been completely eliminated. The PRISMA flow diagram detailing the study extraction process is presented in Figure 1.

### 3.2 Population distribution of COVID-19 infection in China

**Figure 2:**
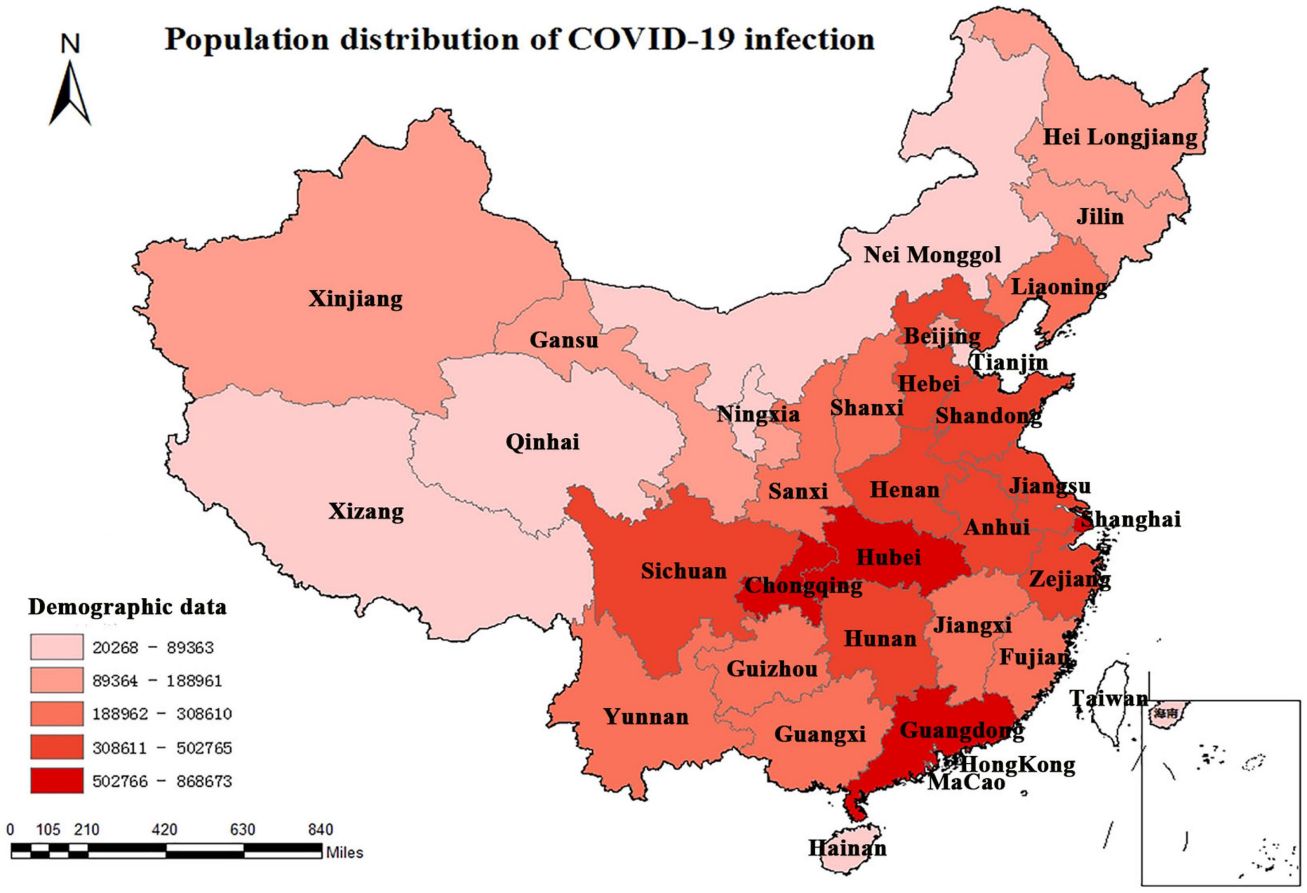
Population distribution of COVID-19 infection in China.

We counted data on the distribution of the infected population since the COVID-19 pandemic in China until 6 December 2022. Data show that COVID-19 caused 9,212,751 confirmed infections in China during the 3 years of the pandemic. Guangdong province won first place with 868,673 infections, 64.5% of the regions had more than 200,000 infections, and more than 71.4% of the infections were concentrated in central and developed coastal cities (Figure 2).

### 3.3 Characteristics of the included articles

**Figure 3:**
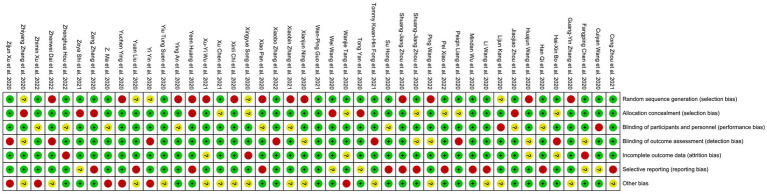
Overall risk of bias assessment in this study.

In total, 44 studies (8, 21–63) with 913,036 participants were included in the meta-analysis, and all studies were cross-sectional surveys according to the qualification criteria. The study population was Chinese citizens, based on males (31.4%) and females (68.6%), with explicit reporting of the prevalence of depression or anxiety in various regions of China during the COVID-19 pandemic. The participant’s occupation types included medical personnel, college students, adolescents, cancer patients, COVID-19 patients, homosexuals, farmers, teachers, pregnant women, and most of the general population. Subject age characteristics were demonstrated as MD ± SD in 14 studies, followed by 8 studies that were explicitly subjected to snowball sampling, and 30 studies reported participant response rates (median 95.1%). A total of 29 studies selected the Patient Health Questionnaire-9-item scale (PHQ-9) to measure depression, while anxiety symptoms were mostly quantified using the Generalized Anxiety Disorder 7-item scale (GAD-7) (in 18 studies). All unclear or unavailable data are indicated as “NR.” Detailed information on baseline characteristics of all studies, including author and year of publication, total number of respondents, participation rate, age, gender (expressed as “%”), region, population type, statistical scales, and outcome indicators (expressed as “%”), are shown in Supplementary File S2. Furthermore, the risk of bias assessment showed 5 studies as high risk (NOS score < 3), while 39 were low-risk studies (NOS score ≥ 3). Details of each NOS scoring are presented in Supplementary File S3, followed by the Cochrane risk of bias assessment results in Figure 3.

### 3.4 Prevalence of depression

**Figure 4:**
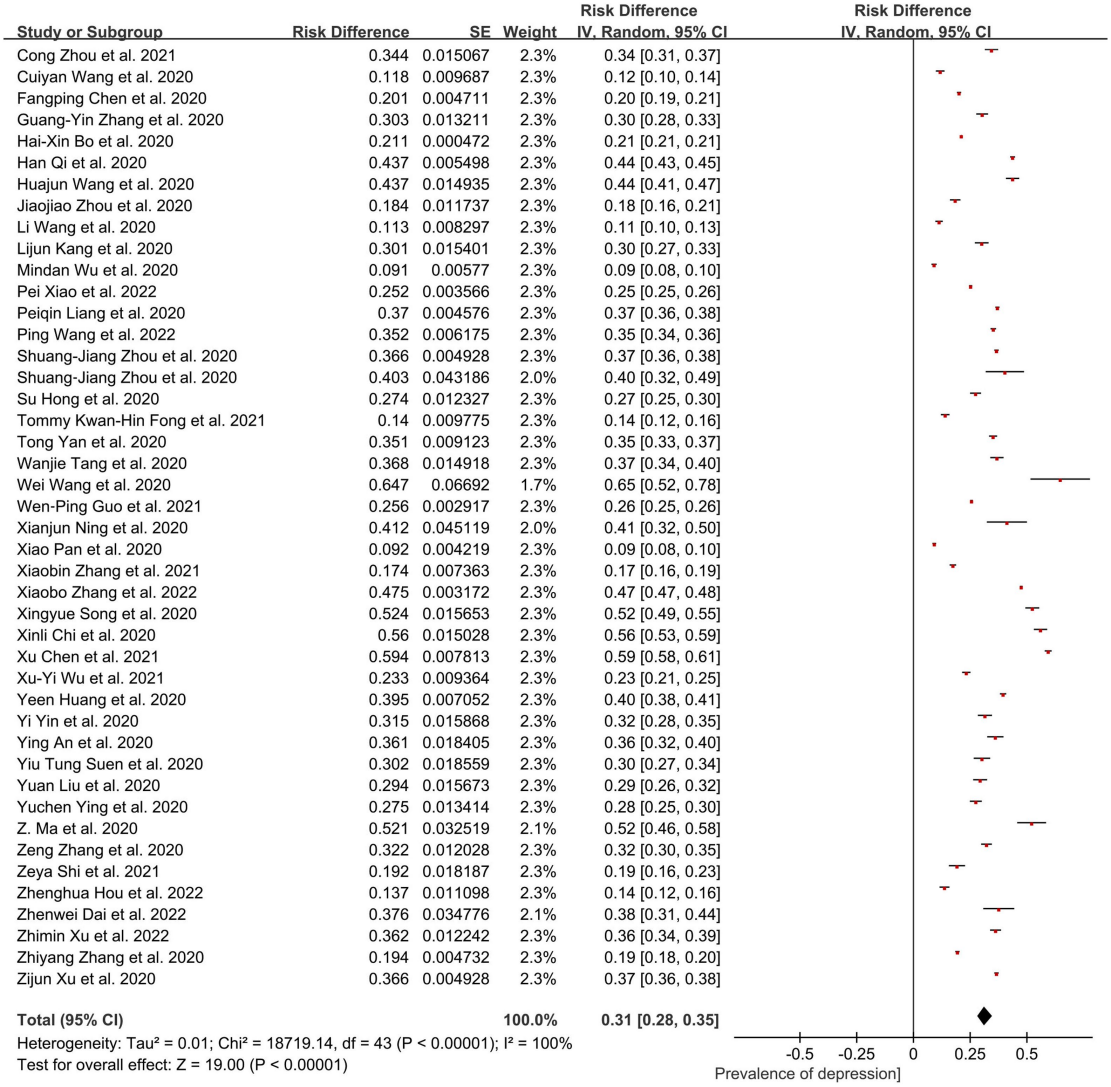
Pooled prevalence of depression in China during the COVID-19 pandemic.

Forty-four studies estimated the prevalence of depression. The ultimate pooled prevalence of depression in Chinese people during the COVID-19 pandemic was 31.0% (95% CI: 0.28–0.35; *Z* = 19; *p* < 0.001), as presented in Figure 4. The significance of the chi-square value and high *I*^2^ statistic (Chi^2^ = 18,719, df = 43, *p* < 0.001; *I*^2^ = 100%) indicated high heterogeneity between studies, whereas the alterations in sensitivity analysis showed that no study affected the pooled prevalence of depression by over 2% [0.18% (8)–1.8% (40)].

### 3.5 Prevalence of anxiety

**Figure 5:**
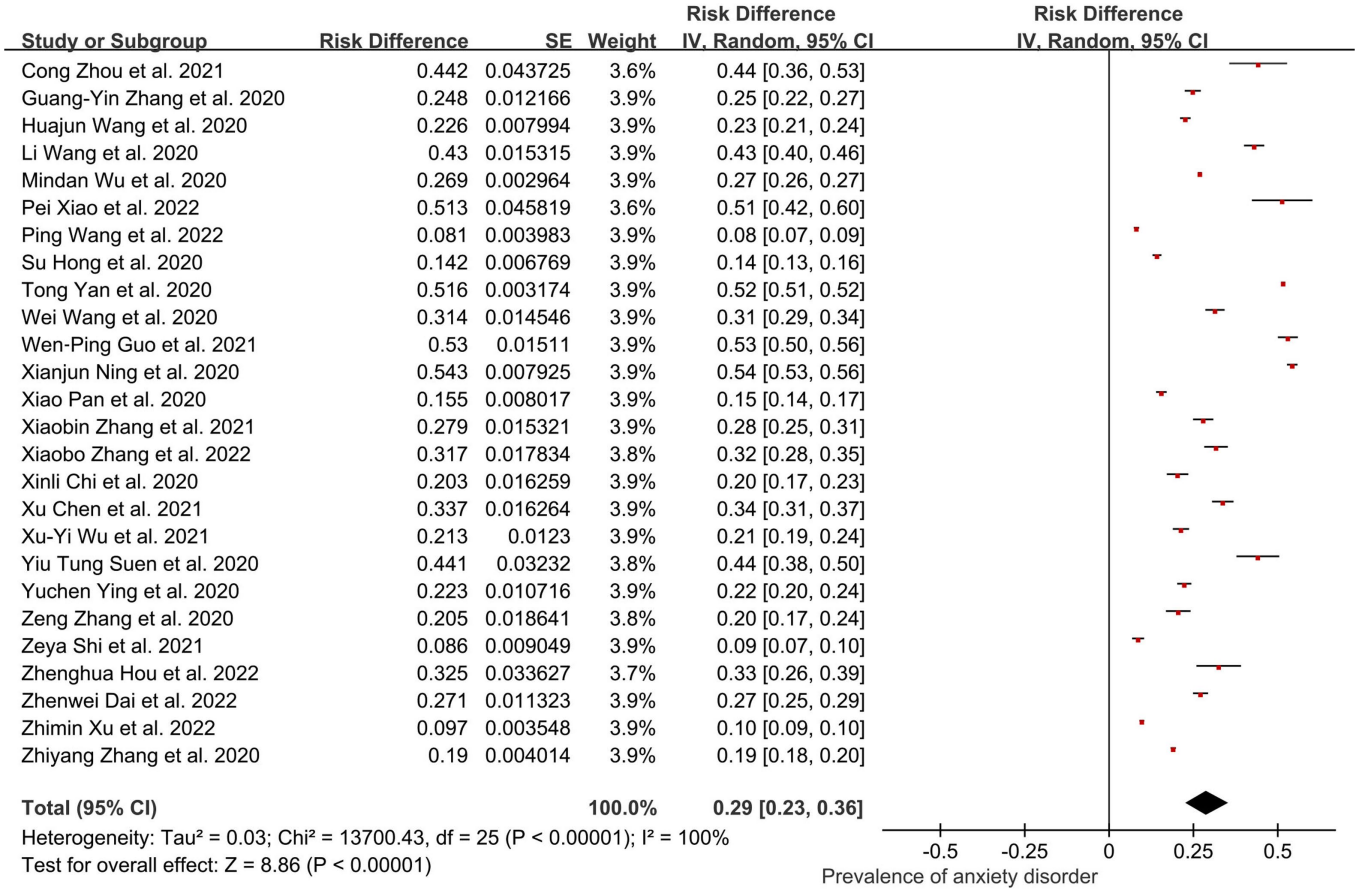
Pooled prevalence of anxiety in China during the COVID-19 pandemic.

In total, 26 of the 44 studies (93,450 participants) reported the prevalence of depression in Chinese people. The corresponding figure for overall anxiety was 0.29 (95% CI: 0.23, 0.36); *I^2^* = 100%, *Z* = 8.86; *p* < 0.001 (Figure 5). After sensitivity analysis, no individual study was found to result in a significant change in the combined prevalence of >2% [0.28% (61)–1.5% (43)].

### 3.6 Subgroup analysis

We only performed subgroup analysis on depression prevalence due to the amount of pooled data for depression that greatly exceeded our expectations and the insufficiency of some vital data for anxiety. In brief, those studies that precisely defined and reported the prevalence of depression in women, students, medical professionals, and adolescents were screened for subgroup analysis.

#### 3.6.1 Women

**Figure 6:**
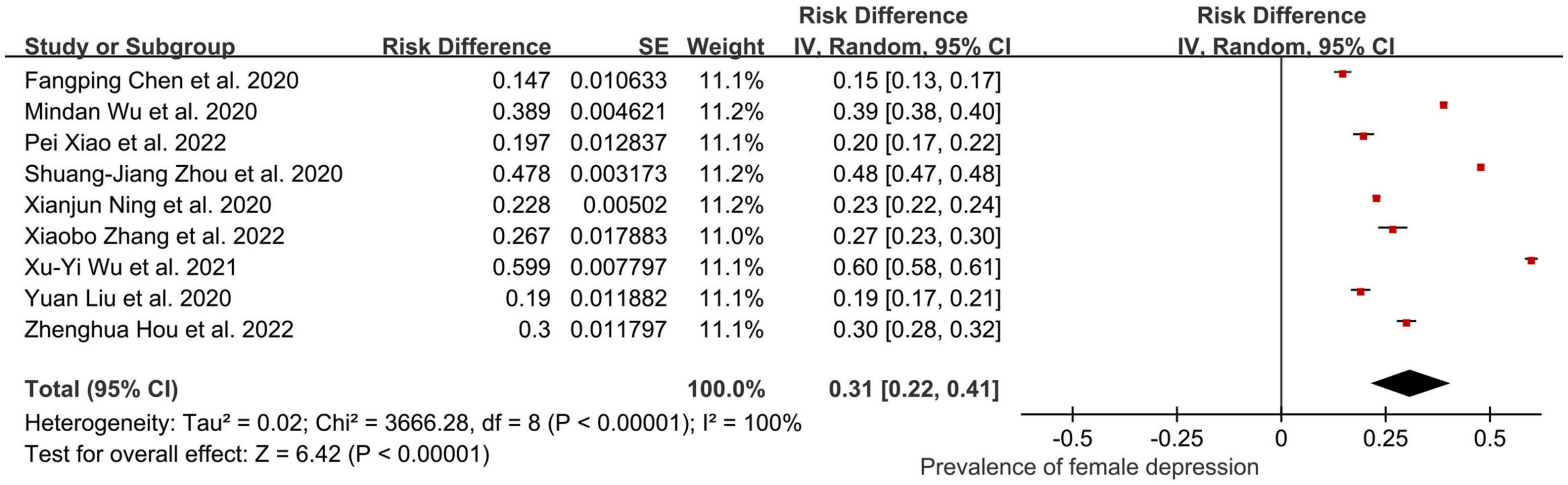
Pooled prevalence of women in China during the COVID-19 pandemic.

Given that women tend to be more sensitive to changes in their circumstances due to COVID-19 and are more likely to experience psychological shock, a subgroup analysis of women was conducted first (45, 48). Subgroup analysis of 9 out of 44 studies (52,137 participants) showed that the final pooled prevalence of female depression was 0.31 (95% CI: 0.22, 0.41); *I*^2^ = 100%, *Z* = 6.42; *p* < 0.001 (Figure 6). After performing the sensitivity analysis, we have found that no study affected the pooled prevalence of depression by over 2% [0.24% (57)–1.78% (48)].

#### 3.6.2 Students

**Figure 7:**
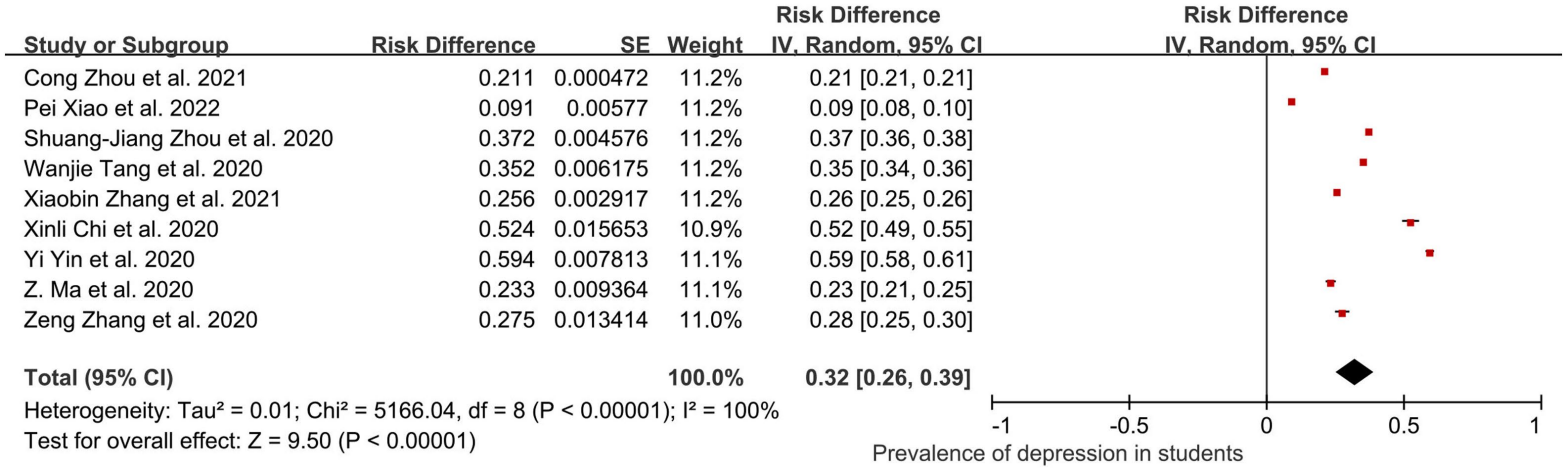
Pooled prevalence of students in China during the COVID-19 pandemic.

In total, 10 of the 44 studies (798,319 participants) were included in subgroup analyses, and these student groups included college and high school students who were accurately defined. The initial student prevalence of comorbid depression was 0.34 (95% CI: 0.25, 0.40); *I*^2^ = 99.80%, *Z* = 10.60; *p* < 0.001. However, a sensitivity analysis of the 10 studies revealed that one study (48) had more than 2% (2.3%) impact on the prevalence of pooled depression. After removing the study, the ultimate prevalence of pooled depression in students was 0.32 (95% CI: 0.26, 0.39); *I*^2^ = 100%, *Z* = 9.50; *p* < 0.001 (Figure 7).

#### 3.6.3 Healthcare workers

**Figure 8:**
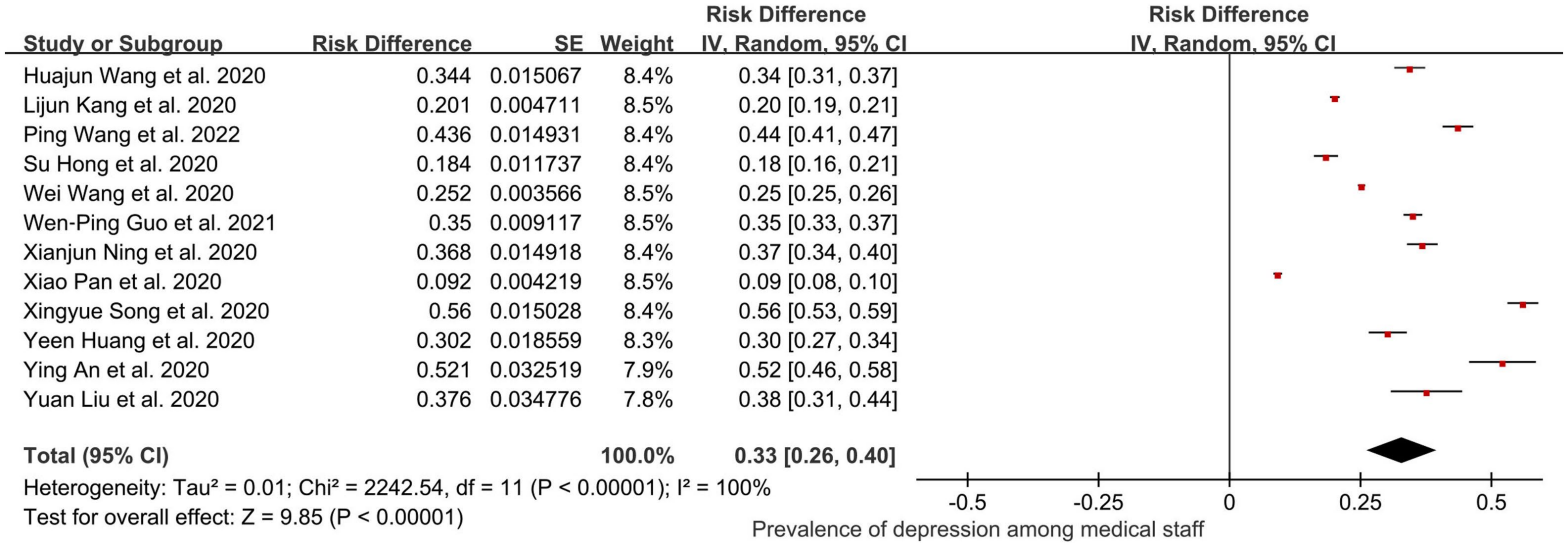
Pooled prevalence of healthcare workers in China during the COVID-19 pandemic.

In total, 12 of the 44 studies (35,855 healthcare workers) were included in subgroup analyses, and all the workers were accurately defined as being in a long-term healthcare profession. The final pooled prevalence of depression among healthcare workers was 0.33 (95% CI: 0.26, 0.40); *I*^2^ = 100%, *Z* = 9.85; *p* < 0.001 (Figure 8). After performing the sensitivity analysis, we found that no study affected the pooled prevalence of depression by over 2% [0.19% (21)–1.98% (43)].

#### 3.6.4 Adolescents

**Figure 9:**
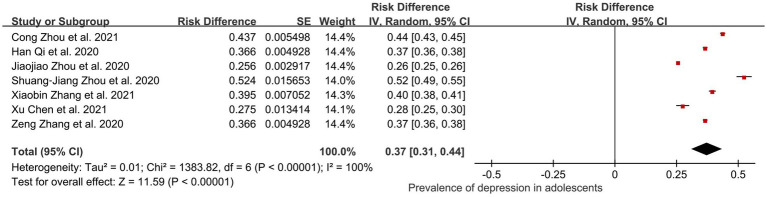
Pooled prevalence of adolescents in China during the COVID-19 pandemic.

Although the international definition of adolescents is based on those between the ages of 10 and 25 years, with some overlap with the student population, the consensus definition of adolescents in China tends to be more vulnerable groups aged 10–19 (64). According to Hawes et al., depression and anxiety symptoms in adolescents and young adults have increased significantly during the COVID-19 pandemic (65). In total, 7 of the 44 studies (56,559 adolescents) were included in the subgroup analysis. The eventual pooled prevalence of depression among adolescents was 0.37 (95% CI: 0.31, 0.44); *I*^2^ = 100%, *Z* = 11.59; *p* < 0.001 (Figure 9). The changes in the estimated pooled prevalence of depression after performing influence analysis ranged from 0.08% (34) to 1.2% (46).

## 4 Discussion

The ravages of COVID-19 have had a serious and far-reaching impact on all aspects of the world, and *The Lancet* has more than once called for increased attention to mental health risks resulting from the epidemic (66–68). As of 2023, COVID-19 had repeatedly ravaged China, infecting 9.21 million residents, especially in the rich central and coastal cities, resulting in an unprecedented disaster for ordinary residents’ livelihoods (Figure 2). Given the potential harm to mental health arising from COVID-19, the present systematic review and meta-analysis estimated the pooled prevalence of depression and anxiety in the Chinese population during the COVID-19 pandemic.

In this meta-analysis of 44 cross-sectional studies, the prevalence of depression and anxiety in the Chinese population is 31 and 29%, respectively, which is generally high. According to Damian Santomauro (69), in the survey of the prevalence of depression and anxiety during the worldwide COVID-19 pandemic in 204 countries and regions in 2020, the prevalence of depression and anxiety in Chinese people was only less than 10.1 and 14.0%, respectively, far lower than that in other developed countries in the world. Surprisingly, this estimate is almost triple of what was previously reported in Bareeqa et al.’s meta-analysis (70) of Chinese people’s mental health at the beginning of 2020, where the prevalence of depression and anxiety disorder was 26.9 and 21.8%, respectively. Similarly, our combined results likewise differ significantly from estimates from Bareeqa et al.’s research in the early stages of the COVID-19 pandemic, whereas they are broadly in line with the findings that the prevalence of depression and anxiety disorders were 31.4 and 31.9%, respectively, during the middle of the COVID-19 pandemic in China (71). As advocated by Shou et al. (72), mental health risks may gradually converge to a stable range with fluctuations in epidemic infectivity and change variously in the general public’s tolerance to the pandemic. Our study is therefore considered to be a comprehensive renovation or complement to previous investigations.

The subgroup analysis of this systematic review and meta-analysis revealed that the prevalence of depression was higher among healthcare workers (0.33), students (0.32), and adolescents (0.37) during the COVID-19 pandemic, which was significantly different from the results of previous reviews (73–75). Given that the public tends to emphasize mental health concerns for minors at the expense of college students as a specific group of youth, as well as to make this study more consistent with the need to screen the scope of a particular study, separate subgroup analyses were performed for adolescents as well as for students. Furthermore, compared to men (0.27 ± 0.03, MD ± SD), the higher prevalence of depression in women (0.31 ± 0.02) during a pandemic may be attributed to the increased domestic violence, fertility burden, and sensitivity to changes in the family economy that may result in more emotional support (76–78). Sensitivity analyses suggested that only a few studies had a tangible impact on the combined prevalence of depression and anxiety, indicating that the results of our meta-analysis are overall reliable. Given the high prevalence of depression among females, adolescents, and healthcare workers, it is essential to take preventive and intervention measures in advance for the appropriate risk groups (79). If timely measures to intervene in mental health are not taken, suicidality is one of the most horrific sequelae of untreated mental illness, and even early in the pandemic, it started to show itself (80).

Therefore, we attempted to comprehensively quantify the prevalence and extent of mental health concerns (depression and anxiety) among residents from the beginning of the COVID-19 outbreak in China to the pandemic. It is worth mentioning that we have included a large number of recent low-risk studies with an unprecedented expansion in the population types and regions included, which effectively supports the reliability of the final results of this meta-analysis. Furthermore, according to the WHO, the overall prevalence of depression and anxiety disorders among Chinese people in 2018 was only 3.87 and 7.62%, respectively, which is significantly different from all the findings we derived, suggesting that COVID-19 poses a significant risk of mental disorders for Chinese people. In addition, we observed some undeniable differences in the numerical impact on mental health between the different stages of COVID-19, which is reflected in the significantly higher prevalence of COVID-19 in the early population than in the late stage, and therefore we labeled the different stages accordingly. Of the 44 articles included, the number of articles in the early, middle, and late stages of the COVID-19 pandemic was 25, 11, and 8, respectively. Our definition of the pandemic stage was defined based on the policy urgency and the scale of prevalence in China. We also recognize several limitations in our meta-analysis. First, studies included in this meta-analysis are all cross-sectional surveys with no randomization, which leads to unavoidably highly heterogeneous results when conducting pooled analysis. Second, due to the limited number of studies containing demographic-type information, no subgroup analysis of anxiety was conducted. Third, due to the insufficient number of current studies, the overall sample size of medical staff is small, which may reduce representativeness. Fourth, although intermittent closure policies may cause or exacerbate a variety of social uncertainties that may also be closely associated with the COVID-19 pandemic, there are still other potential uncertainties that may interfere with mental health, which may reduce representation. Fifth, this study did not analyze prevalence in the middle-aged and older adult population, whereas the COVID-19 pandemic had a more pronounced impact on middle-aged and older adult, which may have resulted in an overestimation of the prevalence values. Furthermore, our study only recorded the psychological health risk data of China as the origin of the epidemic under the COVID-19 pandemic, which may not be universal.

## 5 Conclusion

In conclusion, the prevalence of depression and anxiety among the Chinese was overall high. Monitoring and surveillance of the population’s mental health status during crises such as sudden pandemics are imperative. Although the WHO declared the end of the COVID-19 pandemic, the theoretical results generated during this sudden public crisis could still provide a basis for resolving differences related to the need for population mental health interventions for quite some time and eventually reaching a consensus. Such consensus will also help to address the adverse effects of possible new global public events in future, as we cannot be certain that the emergence of COVID-19 is the end; after all, the SARS coronavirus pandemic is an example.

## Data availability statement

The datasets presented in this study can be found in online repositories. The names of the repository/repositories and accession number(s) can be found in the article/Supplementary material.

## Author contributions

XB: Conceptualization, Data curation, Investigation, Writing – review & editing. K-YQ: Data curation, Investigation, Writing – review & editing. Y-HW: Formal analysis, Validation, Writing – review & editing. LC: Investigation, Validation, Writing – review & editing. Y-JX: Supervision, Validation, Writing – review & editing. JW: Software, Supervision, Writing – review & editing. H-BW: Investigation, Methodology, Writing – review & editing. TB: Project administration, Supervision, Writing – review & editing. C-YD: Writing – review & editing. J-QZ: Conceptualization, Visualization, Writing – original draft.
