# Supplementary material for: Prevalence of depression, anxiety in China during the COVID-19 pandemic: an updated systematic review and meta-analysis
Source: Front Public Health. 2024 Jan 5;11:1267764. doi: 10.3389/fpubh.2023.1267764 (PMC10796455; doi:10.3389/fpubh.2023.1267764)
Supplement: Supplementary file 1 [file Data_Sheet_1.PDF]

Retrieval strategy

#1. Depressive Disorder OR Depressive Disorders OR Disorder, Depressive OR Disorders, Depressive OR Neurosis, Depressive OR Depressive Neuroses OR Depressive Neurosis OR Neuroses, Depressive OR Depression, Endogenous OR Depressions, Endogenous OR Endogenous Depression OR Endogenous Depressions OR Depressive Syndrome OR Depressive Syndromes OR Syndrome, Depressive OR Syndromes, Depressive OR Depression, Neurotic OR Depressions, Neurotic OR Neurotic Depression OR Neurotic Depressions OR Melancholia OR Melancholias Unipolar Depression OR Depression, Unipolar OR Depressions, Unipolar OR Unipolar Depressions

#2. Anxiety OR Angst OR Social Anxiety OR Anxieties, Social OR Anxiety, Social OR Social Anxieties OR Hypervigilance OR Nervousness OR Anxiousness

#3. COVID-19 OR COVID 19 OR SARS-CoV-2 Infection OR Infection, SARS-CoV-2 OR SARS CoV 2 Infection OR SARS-CoV-2 Infections OR 2019 Novel Coronavirus Disease OR 2019 Novel Coronavirus Infection OR 2019-nCoV Disease OR 2019 nCoV Disease OR 2019-nCoV Diseases OR Disease, 2019-nCoVCOVID-19 Virus OR Infection, COVID 19 Virus OR Infection, COVID-19 Virus Infections OR Infection, COVID-19 Virus OR Virus Infection, COVID-19 OR Coronavirus Disease 2019 OR Disease 2019, Coronavirus OR Coronavirus Disease-19 OR Coronavirus Disease 19 OR Severe Acute Respiratory Syndrome Coronavirus 2 Infection OR SARS Coronavirus 2 Infection OR COVID-19 Virus Disease OR COVID 19 Virus Disease OR COVID-19 Virus Diseases OR Disease, COVID-19 Virus OR Virus Disease, COVID-19 OR 2019-nCoV Infection OR 2019 nCoV Infection OR 2019-nCoV Infections OR Infection, 2019-nCoV OR COVID19 OR COVID-19 Pandemic OR COVID 19 Pandemic OR Pandemic, COVID-19 OR COVID-19 Pandemics

#4. China OR Chinese

*Final retrieval strategy: **(#1 OR #2) AND #3 AND #4**
