# Supplementary material for: Prevalence of depression, anxiety in China during the COVID-19 pandemic: an updated systematic review and meta-analysis
Source: Front Public Health. 2024 Jan 5;11:1267764. doi: 10.3389/fpubh.2023.1267764 (PMC10796455; doi:10.3389/fpubh.2023.1267764)
Supplement: Supplementary file 2 [file Data_Sheet_2.pdf]

Table 1. Baseline characteristics of included studies

NO	First author & year published	Epidemic stage	Total number of respondent (N)	Response rate (%)	Age (MD±SD)	Gender (%) Female/Male	Region	Study population	Scales	Depression scale ratings	Anxiety scale ratings	Prevalence of depression (%)	Prevalence of anxiety (%)
1	Lijun Kang et al. 2020	early	994	NR	NR	85.5%/14.5%	Wuhan	Medical staff	PHQ-9	34.4% mean PHQ-9: 8.4	None	34.40%	None
2	Fangping Chen et al. 2020	early	1109	93.40%	NR	48.7%/51.3%	China	General population	DSRS-C	11.78% mean DSRS-C: 19.8	None	11.78%	None
3	Yeen Huang et al. 2020	mid-term	7236	85.30%	35.3 ± 5.6	54.6%/45.4%	China	Medical staff	CES-D	20.1% mean CES-D: 29.5	None	20.10%	None
4	Cuiyan Wang et al. 2020	early	1210	92.79%	NR	67.3%/32.7%	China	College student	DASS-21	30.3% DASS-21: 10-42	None	30.30%	None
5	Z. Ma et al. 2020	early	746217	90.90%	NR	55.6%/44.5%	China	College student	PHQ-9	21.1% mean PHQ-9: 7.7	None	21.10%	None
6	huang-Jiang Zhou et al. 202	early	8140	99.25%	NR	53.5%/46.5%	China	Teenagers	PHQ-9	43.7% PHQ-9: 5.0-17.0	None	43.70%	None
7	Ying An et al. 2020	early	1103	NR	32.20 ± 7.6	90.8%/9.2%	China	Medical staff	PHQ-9	43.61% mean PHQ-9: 9.2	None	43.61%	None
8	Yuan Liu et al. 2020	mid-term	1090	NR	NR	80.2%/19.8%	China	Medical staff	PHQ-9	18.4% PHQ-9: 5.0-18.0	None	18.40%	None
9	Zijun Xu et al. 2020	mid-term	1456	NR	33.8 ± 10.5	59.1%/40.9%	China	General population	PHQ-2	11.3% mean PHQ-9: 4.8	None	11.30%	None
10	Peiqin Liang et al. 2020	early	887	97.41%	NR	100%/0%	Guangzhou	Pregnant woman	EPDS	30.0% EPDS: 14-25	None	30.00%	None
11	Wanjie Tang et al. 2020	early	2485	69.30%	NR	60.8%/39.2%	China	College student	PHQ-9	9.0% mean PHQ-9: 8.2	None	9.00%	None
12	Xingyue Song et al. 2020	early	14825	NR	34.0 ± 8.2	64.3%/35.7%	China	Medical staff	CES-D	25.2% mean CES-D: 28.5	None	25.20%	None
13	huang-Jiang Zhou et al. 202	early	11133	97.90%	NR	62.3%/37.7%	China	College student	PHQ-9	37.0% mean PHQ-9: 7.7	None	37.00%	None
14	Yi Yin et al. 2020	early	5982	NR	NR	60.0%/40.0%	China	College student	PHQ-9	35.20% PHQ-9: 5.0-18.2	None	35.20%	None
15	Han Qi et al. 2020	early	9554	98.05%	NR	52.1%/47.9%	China	Teenagers	CES-D	36.6% mean CES-D: 27.5	None	36.60%	None
16	Guang-Yin Zhang et al. 2020	early	129	NR	43.8 ± 12.6	38.0%/62.0%	China	OVID-19 positive populatio	PHQ-9/GAD-7	40.3% mean PHQ-9: 4.8	44.2% mean GAD-7: 9.5	40.30%	44.20%
17	Hai-Xin Bo et al. 2020	early	1309	NR	NR	100%/0%	China	Pregnant woman	PHQ-9	27.43% PHQ-9: 5.0-17.0	None	27.43%	None
18	Tong Yan et al. 2020	early	1,260	90.97%	NR	43.8%/56.2%	China	Quarantined population	PHQ-9/GAD-7	14.0% PHQ-9: 5.0-18.0	24.8% GAD-7: 5-17	14.00%	24.80%
19	Wei Wang et al. 2020	early	2737	98.60%	NR	64.5%/35.5%	Hubei	Medical staff	HADS-D/HADS-A	35.0% mean HADS-D: 17.6	22.6% mean HADS-A: 16.5	35.00%	22.60%
20	Huajun Wang et al. 2020	early	1045	99.60%	NR	85.8%/14.2%	Guangdong	Medical staff	HADS-D/HADS-A	36.80% mean HADS-D: 15.2	43.0% mean HADS-A: 16.1	36.80%	43.00%
21	Tommy Kwan et al. 2021	mid-term	51	NR	NR	86.3%/13.7%	Hong Kong	Dementia patients	CES-D	64.7% CES-D: 20.0-48.0	None	64.70%	None
22	Xiaobin Zhang et al. 2021	mid-term	22380	97.20%	NR	47.2%/52.8%	Jiangsu	Middle school student	PHQ-9/GAD-7	25.6% PHQ-9: 5.0-17.0	26.9% GAD-7: 5-16	25.60%	26.90%
23	Zhiyang Zhang et al. 2020	early	119	66.10%	40.25 ± 11.5	37.8%/62.2%	Beijing	OVID-19 positive populatio	PHQ-9/GAD-7	41.2% mean PHQ-9: 10.7	51.3% mean GAD-7: 10.0	41.20%	51.30%
24	Su Hong et al. 2020	early	4692	99.01%	NR	96.9%/3.1%	Chongqing	Medical staff	PHQ-9/GAD-7	9.2% mean PHQ-9: 9.7	8.1% mean GAD-7: 8.5	9.20%	8.10%
25	Zeya Shi et al. 2021	mid-term	2651	97.80%	35.91 ± 10.7	78.5%/21.5%	China	General population	HADS-D/HADS-A	17.35% HADS-D: 11.0-23.0	14.15% mean HADS-A: 15.3	17.35%	14.15%
26	Mindan Wu et al. 2020	early	24789	99.40%	NR	46.3%/53.7%	China	General population	HADS-D/HADS-A	47.5% mean HADS-D: 14.9	51.6% mean HADS-A: 14.6	47.50%	51.60%
27	Zeng Zhang et al. 2020	mid-term	1018	99.80%	16.61 ± 1.06	53.5%/46.5%	Shandong	Middle school student	PHQ-9/GAD-7	52.4% PHQ-9: 5.0-19.0	31.4% GAD-7: 5-17	52.40%	31.40%
28	Wen-Ping Guo et al. 2021	mid-term	1091	63.80%	NR	67%/33%	Hubei	Medical staff	PHQ-9/GAD-7	56.0% mean PHQ-9: 9.6	53.0% mean GAD-7: 9.9	56.00%	53%
29	Pei Xiao et al. 2022	Late	3951	96.30%	19.58 ± 1.7	57.6%/42.4%	China	College student	PHQ-9/GAD-7	59.35% PHQ-9: 5.0-19.0	54.34% GAD-7: 5-16	59.35%	54.34%
30	Xinli Chi et al. 2020	early	2038	95.90%	20.56 ± 1.9	62.9%/37.1%	China	College student	PHQ-9/SAS	23.3% mean PHQ-9: 9.7	15.5% mean SAS: 72.1	23.30%	15.50%
31	Jiaojiao Zhou et al. 2020	early	4805	95.10%	NR	100%/0%	China	Teenagers	CES-D	39.5% mean CES-D: 30.2	None	39.50%	None
32	Yiu Tung Suen et al. 2020	early	857	NR	NR	56.1%/33.9%	Hong Kong	Homosexuality	PHQ-9/GAD-7	31.5% mean PHQ-9: 6.7	27.9% mean GAD-7: 7.9	31.50%	27.90%
33	Li Wang et al. 2020	early	681	99.50%	NR	100%/0%	Changzhou	Pregnant woman	PHQ-9/GAD-7	36.12% PHQ-9: 5.0-16.0	31.72% GAD-7: 5-19	36.12%	31.72%
34	Xianjun Ning et al. 2020	mid-term	612	94.20%	NR	72.9%/27.1%	Hunan	Medical staff	SDS/SAS	30.2% mean SDS: 78.4	20.3% mean SAS: 72.9	30.20%	20.30%
35	Yuchen Ying et al. 2020	early	845	95.80%	NR	47.3%/52.7%	Ningbo	General population	PHQ-9/GAD-7	29.35% mean PHQ-9: 6.7	33.73% mean GAD-7: 7.3	29.35%	33.73%
36	Cong Zhou et al. 2021	mid-term	1108	100.00%	16.39 ± 0.8	50.9%/49.1%	China	Middle school student	PHQ-9/GAD-7	27.50% PHQ-9: 5.0-15.0	21.3% GAD-7: 5-17	27.50%	21.30%
37	Ping Wang et al. 2022	Late	236	NR	38.3 ± 8.5	78.4%/21.6%	Shanghai	Medical staff	PHQ-9/GAD-7	52.1% PHQ-9: 5.0-17.0	44.1% GAD-7: 5-17	52.10%	44.10%
38	Xu-Yi Wu et al. 2021	mid-term	1509	93.66%	NR	52.8%/47.2%	China	General population	PHQ-9/GAD-7	32.2% mean PHQ-9: 8.4	22.3% mean GAD-7: 8.9	32.20%	22.30%
39	Zhimin Xu et al. 2022	Late	469	NR	NR	74.6%/25.4%	China	General population	PHQ-9/GAD-7	19.2% mean PHQ-9: 7.5	20.5% mean GAD-7: 7.6	19.20%	20.50%
40	Zhenghua Hou et al. 2022	Late	960	NR	34.33 ± 9.2	39.3%/60.7%	China	OVID-19 positive populatio	PHQ-9	13.7% mean PHQ-9: 7.2	None	13.70%	None
41	Xiao Pan et al. 2020	early	194	97.00%	NR	81.4%/18.6%	China	Medical staff	PHQ-9/GAD-7	37.6% mean PHQ-9: 7.3	32.5% mean GAD-7: 9.0	37.60%	32.50%
42	Zhenwei Dai et al. 2022	Late	1541	NR	NR	57.6%/42.4%	China	General population	PHQ-9/GAD-7	36.2% mean PHQ-9: 6.8	27.1% mean GAD-7: 7.5	36.20%	27.10%
43	Xiaobo Zhang et al. 2022	Late	6984	96.90%	NR	63.7%/36.3%	China	General population	PHQ-9/GAD-7	19.39% PHQ-9: 5.0-16.0	9.74% GAD-7: 5-16	19.39%	9.74%
44	Xu Chen et al. 2021	Late	9554	98.01%	14.06 ± 10.8	52.1%/47.9%	China	Teenagers	CES-D/GAD-7	36.6% CES-D: 20.0-49.0	19.0% GAD-7: 5-16	36.60%	19.00%

"NR" is denoted as the presence of unclear or unavailabledata in the extracted articles.

N = number; MD = mean difference; SD = standard deviation.

PHQ-9 = Patient Health Questionnaire-9; PHQ-2 = Patient Health Questionnaire-2; GAD-7 = Generalized Anxiexy Disorde-7; SAS = Self-Rating Anxiety Scale; SDS = Self-rating depression scale.

CES-D = Center for Epidemiological Studies Depression Scale; HADS = Hospital anxiety and depression scale; EPDS = Edinburgh Postnatal Depression Scale.

DASS-21 = Depression Anxiety Stress Scale-21; DSRS-C = Self-Rating Scale for Depressive Disorder in Childhood.
