# Supplementary material for: Prevalence of depression, anxiety in China during the COVID-19 pandemic: an updated systematic review and meta-analysis
Source: Front Public Health. 2024 Jan 5;11:1267764. doi: 10.3389/fpubh.2023.1267764 (PMC10796455; doi:10.3389/fpubh.2023.1267764)
Supplement: Supplementary file 3 [file Data_Sheet_3.PDF]

Table 2. Modified Newcastle-Ottawa scale for risk of bias assessment

Studies	Representativeness (1)	Size (2)	Comparability (3[illegible]	Outcome (4)	Statistics (5)	Total score
Lijun Kang et al. 2020	1	1	0	1	1	4
Fangping Chen et al. 2020	1	1	0	1	1	4
Yeen Huang et al. 2020	1	1	0	1	0	3
Cuiyan Wang et al. 2020	1	1	1	1	1	5
Z. Ma et al. 2020	1	1	0	1	0	3
[illegible]huang-Jiang Zhou et al. 202[illegible]	1	1	1	0	0	3
Ying An et al. 2020	1	0	0	1	0	2
Yuan Liu et al. 2020	1	1	0	1	1	4
Zijun Xu et al. 2020	1	1	0	1	1	4
Peiqin Liang et al. 2020	1	1	1	1	1	5
Wanjie Tang et al. 2020	1	1	0	1	1	4
Xingyue Song et al. 2020	1	1	1	1	0	4
[illegible]huang-Jiang Zhou et al. 202[illegible]	1	1	0	1	1	4
Yi Yin et al. 2020	1	1	0	1	1	4
Han Qi et al. 2020	1	1	0	1	1	4
Guang-Yin Zhang et al. 2020	1	1	0	1	1	4
Hai-Xin Bo et al. 2020	1	1	1	1	0	4
Tong Yan et al. 2020	1	1	0	1	1	4
Wei Wang et al. 2020	1	0	0	0	1	2
Huajun Wang et al. 2020	1	1	0	1	1	4
Tommy Kwan et al. 2021	1	1	0	1	1	4
Xiaobin Zhang et al. 2021	1	1	0	1	0	3
Zhiyang Zhang et al. 2020	1	1	0	1	0	3
Su Hong et al. 2020	1	1	0	1	0	3
Zeya Shi et al. 2021	1	1	1	1	1	5
Mindan Wu et al. 2020	1	1	0	1	1	4
Zeng Zhang et al. 2020	1	1	0	1	1	4
Wen-Ping Guo et al. 2021	1	1	0	1	1	4
Pei Xiao et al. 2022	1	1	0	1	0	3
Xinli Chi et al. 2020	1	1	0	1	1	4
Jiaojiao Zhou et al. 2020	1	1	0	1	1	4
Yiu Tung Suen et al. 2020	1	1	0	1	1	4
Li Wang et al. 2020	1	1	0	0	0	2
Xianjun Ning et al. 2020	1	1	0	0	1	3
Yuchen Ying et al. 2020	1	1	0	1	1	4
Cong Zhou et al. 2021	1	1	0	1	1	4
Ping Wang et al. 2022	1	0	0	0	1	2
Xu-Yi Wu et al. 2021	1	0	0	1	1	3
Zhimin Xu et al. 2022	1	0	0	1	0	2
Zhenghua Hou et al. 2022	1	1	1	1	1	5
Xiao Pan et al. 2020	1	1	0	1	1	4
Zhenwei Dai et al. 2022	1	1	1	1	0	4
Xiaobo Zhang et al. 2022	1	1	0	1	1	4
Xu Chen et al. 2021	1	1	1	1	1	5
